# Studies of postpartum mammary gland involution reveal novel pro-metastatic mechanisms

**DOI:** 10.20517/2394-4722.2019.01

**Published:** 2019-02-19

**Authors:** Taylor R. Wallace, Sarah E. Tarullo, Lyndsey S. Crump, Traci R. Lyons

**Affiliations:** 1 Department of Medicine, Division of Medical Oncology, University of Colorado Anschutz Medical Campus, Aurora, CO 80045, USA.; 2 Young Women’s Breast Cancer Translational Program, University of Colorado Anschutz Medical Campus, Aurora, CO 80045, USA.; 3 University of Colorado Cancer Center, University of Colorado Anschutz Medical Campus, Aurora, CO 80045, USA.; 4 University of Colorado Gates Center for Regenerative Medicine, University of Colorado Anschutz Medical Campus, Aurora, CO 80045, USA.

**Keywords:** Postpartum involution, postpartum breast cancer, Semaphorin 7a, cyclooxygenase-2, collagen, metastasis

## Abstract

Postpartum involution is the process by which the lactating mammary gland returns to the pre-pregnant state after weaning. Expression of tumor-promotional collagen, upregulation of matrix metalloproteinases, infiltration of M2 macrophages, and remodeling of blood and lymphatic vasculature are all characteristics shared by the involuting mammary gland and breast tumor microenvironment. The tumor promotional nature of the involuting mammary gland is perhaps best evidenced by cases of postpartum breast cancer (PPBC), or those cases diagnosed within 10 years of most recent childbirth. Women with PPBC experience more aggressive disease and higher risk of metastasis than nulliparous patients and those diagnosed outside the postpartum window. Semaphorin 7a (SEMA7A), cyclooxygenase-2 (COX-2), and collagen are all expressed in the involuting mammary gland and, together, predict for decreased metastasis free survival in breast cancer. Studies investigating the role of these proteins in involution have been important for understanding their contributions to PPBC. Postpartum involution thus represents a valuable model for the identification of novel molecular drivers of PPBC and classical cancer hallmarks. In this review, we will highlight the similarities between involution and cancer in the mammary gland, and further define the contribution of SEMA7A/COX-2/collagen interplay to postpartum involution and breast tumor progression and metastasis.

## BREAST CANCER METASTASIS AND POSTPARTUM BREAST CANCER

In the United States, breast cancer remains the second leading cause of cancer related death in women, with the majority of these deaths resulting from metastatic disease. Breast cancer is a heterogeneous disease with five identified molecular sub-types: luminal-A, luminal-B, HER2-enriched, basal-like, and normal-like^[1]^. Treatments for hormone receptor positive and HER2 amplified cases include targeted therapies that, while initially successful, often result in the development of resistance, thereby increasing the likelihood of metastasis^[2]^. Breast cancers lacking estrogen receptor, progesterone receptor, and HER2 amplification are defined as triple negative breast cancers (TNBC) and account for 15%−20% of breast cancer diagnoses. Patients with TNBC not only have lower survival rates due to increased early metastasis, but also lack a targeted therapeutic option^[3,4]^. This evidence underscores the necessity of novel models for the identification of molecular drivers of breast cancer progression, therapy resistance, and metastasis.

Multiple epidemiological studies highlight the effect of pregnancy on breast cancer risk. While first pregnancy at an early age confers a lifelong protective effect against breast cancer, all women who have given birth undergo a transient period of increased risk^[5–9]^. Previously, women diagnosed within five years of most recent childbirth were found to be at higher risk for developing metastatic breast cancer than nulliparous women^[5–12]^, a risk that persists even after adjustments have been made for variability in hormonal receptor status, HER2 status, age, histological grade, tumor size, node status, and year of diagnosis^[10,13]^. More recent results have revealed that the probability of metastasis is increased for women diagnosed with breast cancer between 0–5 and 5–10 years postpartum. Thus, we now define breast cancers diagnosed within 10 years of most recent childbirth as postpartum breast cancer (PPBC)^[10,13]^. By this definition, PPBC accounts for over half of all breast cancers diagnosed in women under age 40 in two independent cohorts^[10,13]^. Since patients with PPBC have significantly worse outcomes, regardless of numerous clinical parameters, it has been hypothesized that pro-tumorigenic changes in the breast tissue following pregnancy may persist for an extended period and accelerate PPBC progression. Consistent with this hypothesis, Asztalos *et al*.^[14]^ identified a breast cancer associated genetic signature in the normal breast tissue of parous women that persists for up to 10 years after childbirth. Further evidence of a postpartum tumorigenic signature is supported by multiple pre-clinical models, where implantation of tumor cells into rodent mammary glands after weaning facilitates tumor cell growth, invasion, and metastasis. Additional studies of the mammary gland after lactation have revealed that these phenotypes are driven, in part, by mammary and tumor specific increases in pro-inflammatory cyclooxygenase-2 (COX-2), fibrillar collagen, semaphorin 7a (SEMA7A), bone marrow derived stromal and macrophage populations, lymphangiogenesis, and circulating estrogens^[15–22]^. In this review, we will explore some of the mechanisms by which post-lactational changes in the mammary gland facilitate breast cancer progression and metastasis, with a focus on the roles of collagen, COX-2, and SEMA7A in cell death, extracellular matrix (ECM) and vascular remodeling, and macrophage infiltration.

## PRO-TUMORIGENIC ROLES OF INVOLUTION ASSOCIATED PROGRAMS

For a detailed review of the events that occur during embryonic and adult mammary gland development, see Macias and Hinck^[23]^. Briefly, mammary gland development begins during embryogenesis where ectodermal placodes invade into the mammary mesenchyme to form a rudimentary ductal tree^[24]^. This primitive structure persists until puberty where upregulation of growth hormone and estrogen coordinate ductal morphogenesis to form the extensive epithelial ductal network that fills the mammary fat pad. Mammary epithelial structures are bi-layered, meaning they consist of both luminal cells and basally restricted myoepithelial cells that contact the basement membrane^[25]^. Full differentiation of the mammary gland is not achieved until pregnancy, where progesterone and prolactin coordinate differentiation of the alveolar structures that are responsible for milk storage and secretion; in addition, continued branching morphogenesis occurs to prepare the gland for lactation^[23,26]^. During lactation, oxytocin stimulates the contraction of myoepithelial cells in response to suckling, resulting in milk delivery via the nipple^[27]^. When lactation ends, the gland must cease milk production and return to the pre-pregnant karchitecture through postpartum involution.

Postpartum involution involves the upregulation of tumor-promotional factors in the mammary epithelium and surrounding stroma. As primary components of the mammary ECM, collagen proteins, and the fibroblasts that produce and remodel them, have been extensively studied for their role in breast tumor progression and metastasis^[28,29]^. The 28 types of collagen proteins share a triple α-helix as part of their structure, and they can be broadly classified as fibrillar or non-fibrillar based on their assembly^[30]^. For additional information on collagen and its assembly, see Mouw *et al*.^[30]^. In this review, we will focus on fibrillar collagen, as this is the most abundant form in the mammary gland^[31]^. Collagen I expression is both spatially and temporally regulated, and its tight control is essential for the proper mechanosignaling required for normal mammary gland development and function^[31]^. In fact, dysregulation of the primary collagen I receptor, α_2_β_1_ integrin, can alter mammary ductal branching and promote tumor formation^[32,33]^. The contribution of collagen to tumor progression is emphasized by the prognostic value of collagen I mRNA in clinical outcomes of breast cancer^[34]^ and evidence that increased collagen density promotes local breast cancer invasion and distant metastasis^[35]^. One way that collagen deposition is regulated during involution is by the pro-inflammatory enzyme COX-2.

COX enzymes were first identified as the targets of inhibition by aspirin and other non-steroidal anti-inflammatory drugs (NSAIDs)^[36]^. COX enzymes exist in two isoforms, both of which bind to cell membranes where they catalyze the metabolism of free arachidonic acid (AA) to prostaglandins (PGs) via phospholipase A2^[37,38]^. First, COX enzymes rapidly catalyze the formation of an unstable intermediate, prostaglandin G2 (PGG2), from AA. PGG2 is then rapidly converted to prostaglandin H2 (PGH2) via COX-mediated peroxidase activity. Finally, specialized prostaglandin synthases result in the conversion of PGH2 to specific, biologically active PGs^[37–39]^. Though initially believed to function identically, later investigations revealed important differences between COX-1 and COX-2^[40]^. COX-1 is constitutively expressed as a regulator of tissue homeostasis^[41]^, whereas COX-2 is not normally expressed in adult tissues, with the exception of the central nervous system^[42]^, kidneys^[43]^, and male reproductive organs^[40,44]^. Unlike COX-1, COX-2 expression is regulated by mitogens, hormones, and cytokines, and it is also correlated with cancer progression^[45,46]^. However, it is the product of COX-2 activity - PGE2 - that serves as the active effector of pro-tumorigenic signaling. Once synthesized, PGE2 can bind to specific E-prostanoid receptors on the cell surface to activate pathways associated with survival and inflammation^[47]^. COX-2/PGE2 signaling has been described in multiple models of cancer, including oral, breast, prostate, and colorectal, with documented roles in tumor initiation, invasion, immune evasion, cell survival, metastasis, vascular remodeling, cancer stem cells, and drug resistance; for further review, see Hashemi Goradel *et al.*^[46]^ and Stasinopoulos *et al*.^[48]^. In addition to the feedback between COX-2 and collagen deposition in involution and in breast cancer^[18]^, we have also published a connection between COX-2 and tumor cell invasion through expression of the neuronal guidance protein, SEMA7A^[49]^.

SEMA7A, or CD108w, was first recognized for its expression on lymphocytes^[50]^; however, a role for SEMA7A in cancer was later identified through its association with Plexin-C1, the receptor for the viral homolog of SEMA7A. Engagement of Plexin C1 by viral semaphorin inhibits dendritic cell adhesion and motility via alterations to the actin cytoskeleton^[51]^. Similarly, in melanoma, SEMA7A/Plexin-C1 engagement inhibits cell migration via inactivation of the cofilin pathway^[52]^. Cofilin activation, which normally generates free actin filaments required for cell migration^[53]^ is considered a major component of the metastatic cascade^[54]^. SEMA7A/Plexin-C1 mediated cofilin inactivation led to the identification of Plexin-C1 as a novel tumor suppressor^[53]^. Consistent with this finding, Plexin-C1 expression is frequently lost in melanoma^[53]^. Conversely, during development, SEMA7A signals through its other known receptor, β1-integrin, to promote axon outgrowth via activation of the mitogen activated protein kinase (MAP-K) pathway^[55]^. Unlike Plexin-C1, SEMA7A-mediated activation of β1-integrin has been shown to promote cancer progression. Our lab and others have shown that SEMA7A-β1-integrin binding promotes cell migration, invasion, metastasis, and neo-vasculogenesis of the blood and lymphatic vessels^[49,56–58].^ SEMA7A has also been implicated in multiple models of fibrosis (reviewed below), supporting additional roles in inflammation and fibrillar collagen deposition. The expression of SEMA7A, COX-2, and collagen during postpartum involution and their known roles in facilitating tumor progression suggest these molecules and their interplay may be important drivers of PPBC.

## PRO-TUMORIGENIC MECHANISMS OF CELL DEATH DURING INVOLUTION

Programmed cell death is essential for preventing the aberrant cellular phenotypes that arise when cells acquire DNA damage and mutations. Resistance to cell death may, consequently, result in the propagation of potentially harmful mutations that allow cells to bypass checkpoints meant to prevent unregulated growth and division. Resistance to cell death is thus defined by Hanahan and Weinberg^[59]^ as one of the original hallmarks of cancer. For a detailed review of cell death and cancer, see Ichim and Tait^[60]^. In tumor cells, resistance to cell death plays an important role beyond tumor initiation, affecting drug-resistance, recurrence, and metastasis. Despite continuous advances in targeted chemotherapy, treatment efficacy tends to decline over time as a result of the ability of tumor cells to suppress apoptotic pathways, upregulate DNA repair, and genetically adapt to escape death. Tumor cells with repopulating capacity may also acquire the ability to resist death in response to chemotherapy, thereby constituting a major mechanism of cancer recurrence. Mechanisms by which tumor cells resist chemotherapy are further summarized by Al-Dimassi *et al*.^[61]^. Active survival signals from the mammary ECM are required to override the default programming of normal adherent cells to die. When these cells become detached, they die in the absence of ECM signaling by a specialized form of programmed cell death called anoikis^[62]^. Anoikis-resistance is a critical requirement for the successful metastasis of circulating tumor cells, a feature that may be shared by some of the cells that survive involution^[63]^. Postpartum involution is characterized by two major waves of apoptosis, culminating in the death of over half of the mammary epithelium^[64]^. As a minority of mammary epithelial cells (MECs) survive this process, postpartum involution represents an excellent model for studying the mechanisms by which cells acquire resistance to cell death in apoptotic environments. In this section, we will review the major mechanisms regulating cell death during postpartum involution and potential contributors to cell death resistance during tumorigenesis.

Postpartum involution occurs in two primary phases. In the first, or reversible phase, accumulation of milk protein, a process known as milk stasis, results in the detachment and shedding of secretory alveolar epithelial cells to the lumen. Milk stasis induces the first wave of cell death, in part, through the local expression of leukemia inhibitory factor (LIF). LIF is a primary activator of the master apoptotic regulator of involution-signal transducer and activator of transcription 3 (STAT3)^[65]^. Other important activators of STAT3 at the onset of postpartum involution include transforming growth factor-β (TGFβ), Janus kinase-1 (JAK-1), and Snail2 (SLUG)^[66–68]^. Activation of STAT3 promotes postpartum mammary gland involution by shifting the balance of pro-and anti-apoptotic signals in favor of programmed cell death. STAT3 coordinates the first wave of epithelial apoptosis through activation of pro-apoptotic Bcl-2 family members, upregulation of PI3K inhibitory subunits, and downregulation of MAP-K survival signaling^[66]^. STAT3 further promotes cell death during involution by mediating the formation of triglyceride-containing vacuoles. Triglycerides within these lysosomal-like vacuoles become metabolized into free fatty acids which interact with and distort lysosomal membranes. This results in the leakage of cathepsin proteases into the cytosol, and ultimately, lysosomal-mediated programmed cell death^[69]^. The role of STAT3 in regulating apoptosis during involution has recently been comprehensively reviewed by Hughes and Watson^[66]^. STAT3 has a well-defined role in cancer progression, with over 40% of breast cancers presenting with constitutive STAT3 activation. Because STAT3 activates multiple signaling pathways, aberrant activation can promote multiple changes associated with cancer, including altered cell cycle dynamics, EMT, angiogenesis, and interestingly, resistance to cell death^[70,71]^. Therapies targeting STAT3 may, thus, be of important therapeutic value for PPBC patients. For more about the role of STAT3 in breast cancer, see Segatto *et al*.^[70]^.

Removal of the overwhelming number of dead cells from the gland requires the Rac-1 mediated switch of MECs from a secretory to a phagocytic phenotype - a process which is essential for proper remodeling in the second phase of involution^[72]^. Despite the massive wave of cell death that occurs during the first phase, if suckling resumes within this window (48–72 h, in mice), involution can be reversed, and lactation can proceed. This reversibility is due, in large part, to the expression of tissue inhibitors of metalloproteinases (TIMPs)^[73]^. TIMPs preserve the reversibility of gland regression by delaying irreversible tissue remodeling events through the inhibition of matrix metalloproteinases (MMPs)^[74]^. Interestingly, TIMP expression may also serve as an additional mechanism to regulate epithelial apoptosis, as TIMP3 has been implicated in the regulation of cell death in a tumor necrosis factor (TNF)-dependent manner^[75]^. The number of compensatory mechanisms that exist to activate apoptosis underscore the importance of postpartum involution to future rounds of successful lactation. At approximately day three of involution in mice, downregulation of TIMPs results in activation of the MMPs that degrade the mammary ECM, causing MECs to lose contact with their underlying basement membrane^[73]^. In the absence of pro-survival signaling from the ECM, detached MECs die by anoikis, thus comprising the second wave of cell death^[62,73]^. Similar to the first phase, clearance of dead cells during the second phase of involution is largely mediated by phagocytic MECs, with additional limited support from professional phagocytes. Milk fat globule epidermal growth factor 8, which works by recognizing phosphatidylserine on the outer leaflet of the plasma membrane of dying cells, is also critical for apoptotic cell clearance during the second phase^[76]^.

What allows some cells to die and others to live during involution remains largely unanswered. To date, more than 50 mammary specific knockouts have been generated that exhibit alterations in postpartum involution - either delayed or premature - that are consistent with the proposed role for each molecule in the process. For example, activation of pro-survival pathways, such as Akt1, and/or deletion of death inducing genes, such as Bax, results in delayed involution, while deletion of Akt1 and anti-apoptotic Bcl-x results in premature involution^[77–80]^. See Radisky *et al*.^[80]^ for an extensive review of these models. As pro-survival signals progressively decline and pro-apoptotic signals increase during the first phase of involution, the mammary basement membrane and ECM become the primary mediators of cell survival^[81]^. Yet, during the second phase, the basement membrane and ECM are degraded by proteases. Previously, SEMA7A mRNA expression was shown to increase in whole mammary extracts during the early phase of involution, and its expression was attributed to its immunomodulatory role^[82]^. Recently, we published that SEMA7A is expressed on Epcam+ MECs during the remodeling phase of involution^[57]^; however, the downstream mechanisms activated by epithelial SEMA7A are not well understood. Interestingly, SEMA7A is a ligand for β1-integrin, which is the receptor for ECM molecules that normally facilitate epithelial cell attachment. It is therefore possible that SEMA7A may activate β1-integrin signaling and provide a pro-survival mechanism to overcome anoikis. Furthermore, β1-integrin mediates the activation of known survival pathways including MAP-K and AKT, which affects survival via stabilization of NF-κB and expression of COX-2. Thus, SEMA7A may promote anoikis-resistance during involution via activation of β1-integrin in a manner that is independent of ECM; however, additional investigation is required.

The role of COX-2 during involution has also largely been attributed to its role in the modulation of the immune milieu^[83]^. Yet, COX-2 also contributes to cell survival mechanisms and collagen remodeling via PGE2, which promotes cell growth and proliferation, modulates collagen expression levels, and upregulates proteinase expression. Studies investigating the effects of postpartum involution in TNBC progression have revealed a feed-forward mechanism by which fibrillar collagen deposition during involution requires COX-2 expression. COX-2 further promotes increased fibrillar collagen^[18]^, which is directly associated with tumor progression and poor prognosis in breast cancer patients^[31,84]^. Cooperation between fibrillar collagen and COX-2 may therefore contribute to the pro-tumorigenic nature of the involuting mammary gland. Similar to postpartum involution, wound healing is a biological process where cell death is accompanied by collagen remodeling. In the “phoenix rising” model of cell death, discovered in a model of wound healing and further characterized in cancer, dying cells send signals to stem and progenitor cells to increase their proliferation, thereby coordinating cell growth with death^[85,86]^. In dying cells, activation of caspases 3 and 7 results in the release of calcium-independent phospholipase-2, which increases the production and release of AA from cell membranes. Ultimately, COX-2 and PGE2 synthase convert free AA to PGE2, which increases stem and progenitor cell proliferation to promote tissue regeneration^[85]^. Though the phoenix rising model, to our knowledge, remains uninvestigated during mammary involution, there are numerous similarities between the wound healing program and postpartum involution, including apoptosis, clearance of damaged and dead cells, and activation of similar inflammatory programs. This suggests that this mechanism of coordinated cell death and tissue regeneration may be conserved in mammary development. Additionally, these mechanisms seem to be particularly important for the growth and survival of stem and progenitor cells in response to apoptotic stimuli, thus representing a mechanism of tumor recurrence in response to chemotherapy. The preceding data may help to further explain the role of collagen, COX-2, and SEMA7A in facilitating tumor progression following lactation.

## EXTRACELLULAR MATRIX AND REMODELING DURING POSTPARTUM INVOLUTION AND CANCER

In their updated review of the hallmarks of cancer, Hanahan and Weinberg described the tumor microenvironment (TME) as a critical mediator of cancer progression due to its ability to supply cancer cells with signals that promote inflammation, induce angiogenesis, and confer resistance to cell death^[87]^. The TME consists of all the cells, ECM molecules, vasculature, and proteins that surround a tumor. The cross-talk between these components and the tumor can affect tumor growth, survival, metabolism, metastasis, response to treatment, and recurrence. The contributions of the TME to cancer progression are comprehensively reviewed in a recent special issue of Nature Reviews Cancer^[88]^. In this section, we will briefly discuss some of the similarities between the tissue microenvironment of the involuting mammary gland and the TME.

ECM fragments, which can be used as diagnostic markers of disease, are known contributors to cancer for their ability to participate in cell signaling events and modulate gene expression^[31,89]^. An investigation of the tumor-promotional aspects of involution revealed that tumor cells co-cultured with ECM isolated from involuting rat mammary gland promoted tumor cell invasion, whereas tumor cells co-cultured with ECM isolated from nulliparous rat mammary glands did not^[90]^, suggesting that involution-derived ECM promotes tumor cell invasion and metastasis. In further support, orthotopic injection of breast cancer cells mixed with ECM isolated from the mammary glands of rats undergoing postpartum involution also confirmed that involution derived ECM promotes metastasis^[90]^. Additional investigations have confirmed that the involution microenvironment mirrors the typical breast TME based on their shared availability of ECM fragments, dramatic increases in fibrillar collagen, and increased MMP activity ^[90–92]^. Specifically, studies of stomelysin-1 (MMP3), the primary active MMP during the tissue remodeling phase of involution, have characterized it as a potent mediator of EMT and other early oncogenic events in the mammary gland^[93]^. Furthermore, elevation of fibrillar collagen results in increased collagen crosslinking and ECM stiffening - characteristics known to accompany breast tumor progression^[89,94–96]^. Increased collagen deposition and cross-linking further promote tumor cell invasion and metastasis by providing a structural network for tumor cell migration^[97]^.

Transforming growth factor β (TGFβ), well-known for its paradoxical role in breast tumor progression, is a primary regulator of collagen deposition in the mammary gland^[98]^. At approximately 8 h post weaning, TGFβ becomes activated and exerts tumor suppressive effects via activation of apoptotic programming^[73,99]^. Conversely, when normal cells begin to take on tumorigenic phenotypes, TGFβ promotes cancer progression by enhancing tumor cell survival and contributing to the maintenance of cancer stem cell populations^[100]^. TGFβ is also essential for wound healing, where it stimulates ECM deposition via fibroblast activation^[101]^. Interestingly, TGFβ dependent fibroblast activation during involution may be COX-2 dependent, as NSAID treatment decreases fibroblast activation *in vivo*. Furthermore, NSAIDs inhibit fibroblast mediated fibrillar collagen deposition during involution and in a model of PPBC^[15]^. Additionally, TGFβ is known to positively regulate SEMA7A in pulmonary fibrosis, where SEMA7A is critical for ECM deposition via activation of the PI3K/AKT pathway^[102]^. Given its role in ECM deposition, SEMA7A expression during the second phase of involution suggests it may also play a role in mammary gland tissue remodeling^[57]^. SEMA7A can affect ECM remodeling through its ability to recruit fibroblasts and immune cells to fibrotic sites, and it has been further implicated in fibrosis models in the liver, kidney, and in glial scar formation^[103–108]^. Contradictory to this role, when endogenously expressed on fibroblasts, SEMA7A can maintain fibroblast homeostasis and reduce pro-fibrotic markers^[109]^, indicating context dependent roles for SEMA7A-mediated signaling. In cancer, fibrillar collagen coordinates upregulation of COX-2 on tumor cells, further promoting tumor cell invasion and metastasis^[7]^, and we have published that COX-2 drives SEMA7A expression^[49]^. Thus, fibrillar collagen, COX-2, and SEMA7A may be a part of a feed-forward loop that ultimately results in cancer cell invasion and metastasis. Additional studies, however, are needed to better understand the hierarchy and cross-talk between these molecules in the context of postpartum involution and breast cancer progression.

Altering fibrillar collagen deposition provides a route for tumor cell migration and invasion, but also changes the signals received by cells from the surrounding environment. Signals from the ECM are communicated to cells by integrins. Integrins are heterodimeric (αβ) transmembrane receptors with 18 known α and 8β integrin subunits, resulting in 24 possible heterodimeric integrin receptors^[110]^. Proper expression and signaling of integrins is essential for cell survival and adhesion, and integrin dysregulation can promote cancer via activation of pathways that affect survival, EMT, and migration^[28,111–115].^ Specifically, improper integrin signaling and expression can result in the loss of normal epithelial cell polarity and attachment, in addition to the over-activation of focal adhesion kinase and subsequent downstream signaling pathways that promote cell survival, migration, invasion, and ultimately metastasis^[116]^. While some integrin pairs are highly specific in their substrate recognition, others can recognize a number of substrates from the ECM, as well as foreign molecules such as snake venom, viral particles, and pathogens^[117,118]^. Alternative ligand-binding partners and/or differential integrin expression can elicit different signaling pathways; thus, when either ligand or integrin profiles are altered, cellular signaling pathways can become aberrantly activated or inhibited. Abnormal SEMA7A expression during involution and/or cancer may promote tumorigenesis via activation of β1-integrin and downstream pathways. SEMA7A binds to β1-integrin via the RGD binding site located on the SEMA domain; however, this site is buried in the crystal structure when SEMA7A is bound to Plexin C1. The ability to differentially regulate tumor progression could, therefore, be explained by binding of SEMA7A to its different receptors^[119,120]^. Further, while SEMA7A binds to α1β1-integrin on inflammatory macrophages^[121]^, the α-binding partner needed for SEMA7A-mediated breast cancer progression is unknown. As reviewed above, one consequence of SEMA7A-β1-integrin signaling is fibrillar collagen deposition. Collagen can also activate integrins and modulate their associated signaling pathways, primarily through α2β1 integrin, which is often upregulated on cancer cells of epithelial origin^[122]^. Collagen binding to α2β1 integrin on tumor cells promotes cellular invasion, which helps cells navigate through the collagen I rich mammary TME and distant metastatic sites, such as the bone^[122]^. Interestingly, α2β1 integrin has been shown to increase COX-2 expression in intestinal epithelial cells^[123]^, leading to activation of downstream signaling events associated with tumor promotion. While these results further support a link between COX-2 and collagen, α2β1 integrin has also been recognized as a metastasis suppressor in breast cancer. The complex interplay between integrins, collagen, COX-2, and SEMA7A is, thus, likely to be context-dependent, and additional studies are necessary to understand the role of this signaling axis in mediating tumor cell invasion and metastasis in breast cancer.

## MACROPHAGES IN POSTPARTUM INVOLUTION AND PPBC

Macrophages are the phagocytic immune cells that mediate the removal of foreign pathogens, dead cells, and debris. Classically-activated macrophages, also known as M1 macrophages, are activated in response to pathogen-associated cytokines, most often IFN-γ and lipopolysaccharide. M1 macrophages are largely considered to be “anti-tumor” based on their expression of the pro-inflammatory cytokines, interleukin-1 (IL-1), IL-12, TNF-α, and inducible nitric oxide synthase - all of which have been shown to oppose tumor progression^[124,125]^. In contrast, Th2 family cytokines induce the maturation of alternatively-activated, or M2 macrophages, and cause their release of anti-inflammatory mediators that support tumor cell survival^[21,126]^. M2 macrophages promote tumor cell growth, invasion, and metastasis, via their secretion of IL-10, TGFβ, and MMPs. Though the M1/M2 system is useful for broadly classifying macrophages, this taxonomy fails to capture the plasticity and diversity characteristic of this cell type. For the purpose of this review, however, we will use M1 and M2 to broadly classify anti-and pro-tumor macrophages, respectively. Multiple studies support the role of macrophages as critical mediators of metastasis^[21,127–129].^ In models of gastric and breast cancer, M2 macrophages are recruited by tumor cells, where they activate MAP-K signaling to promote the motility of disseminating tumor cells^[127]^. Further, macrophages appear to be critical for the migration of the majority of ductal carcinoma *in situ* cells, as only 10% are motile when macrophages are absent^[130]^. The critical contribution of macrophages to tumor cell metastasis is further evidenced by studies in the MMTV-PyMT mouse model of breast cancer, where knockout of colony stimulating factor-1 (CSF-1), a secreted glycoprotein that induces the differentiation of hematopoietic stem cells to macrophages, correlates with a near complete elimination of tumor cell metastasis^[21,131]^. Macrophages, therefore, represent a diverse population of cells that can promote or inhibit tumor progression based on the context of their environment.

Macrophages are the primary immune cells present during mammary gland postpartum involution, and because of their role in facilitating tumor metastasis, represent a potential contribution to the highly metastatic nature of PPBC. Though known primarily for their phagocytic capacity, macrophages only play a minimal role in the clearance of apoptotic cells during involution^[21,132,133]^. Despite their limited role in phagocytosis, M2 macrophages are essential for the epithelial apoptosis and tissue remodeling characteristic of postpartum involution^[134]^. At the peak of apoptotic cell clearance, macrophages exist at relatively low levels, as MECs represent the primary phagocytes. At day 6 of involution, however, the peak of mammary tissue remodeling, M2 macrophages exist at 6 times the level of those in the nulliparous mammary gland, while classically-activated M1 macrophages remain at consistent levels throughout pregnancy, lactation, and gland regression^[21]^. F4/80, a general marker of mature mouse macrophages, marks more than the sum of M1 and M2 macrophages during involution, suggesting there are additional macrophage populations present in the involuting mammary gland^[20]^. Our lab has recently identified a population of macrophages that also express the lymphatic endothelial marker, podoplanin (PDPN)^[57]^. In culture, SEMA7A drives the expression of PDPN on macrophages and promotes their migration and adherence to lymphatic vessels^[57]^. Because macrophages have proven to be a critical part of the metastatic cascade by facilitating intravasation into tumor associated blood vessels ^[135]^, SEMA7A-mediated macrophage lymphatic mimicry may also facilitate intravasation into lymphatic vessels, providing another explanation for the high rates of metastasis associated with PPBC. This is further supported by the prognostic value of a combined genetic signature of CD68, PDPN, and SEMA7A in predicting decreased distant metastasis free survival in a cohort of 600 human breast cancer cases^[57]^. SEMA7A further regulates macrophages by serving as a strong activation factor for monocytes, promoting both chemotaxis and secretion of inflammatory cytokines, in addition to upregulation of granulocyte-macrophage CSF (GM-CSF), supporting an additional role for SEMA7A in macrophage differentiation^[136]^.

Another important macrophage regulator during postpartum involution and breast cancer is COX-2. Previous studies in breast cancer models have shown that COX-2 expression increases with cancer stage, and its expression levels can indicate breast cancer progression, recurrence, and metastasis^[137]^. Recently, COX-2 expressing tumor associated macrophages (TAMs) have been shown to promote the metastatic potential of breast cancer cells via secretion of IL-6 and subsequent activation of AKT signaling in cancer cells^[138]^. Further, expression of COX-2 in stromal TAMs results in upregulation of COX-2 in breast cancer cells, thereby shifting polarization of local macrophages toward the M2 phenotype. In addition to its association with tumor promotional CD163+ TAMs, COX-2 expression in the stroma is further associated with increased collagen alignment in invasive breast cancer^[139]^. TAMs are known to associate with dense regions of collagen in breast cancer in the same way M2 macrophages associate with fibrillar collagen during involution. In the MMTV-PyMT model, macrophages associated with fibrillar collagen have been shown by intravital imaging to migrate across collagen fibers, suggesting that one mechanism by which macrophages promote metastasis is by supporting the migration of tumor cells across collagen networks^[21,140]^. SEMA7A, COX-2, and collagen all represent important effectors of macrophage-mediated tumor cell growth, survival, and metastasis. As macrophages are considered essential for successful metastasis, targeting the molecules responsible for alternative macrophage activation, survival, and chemotaxis may be critical for the successful treatment of metastatic disease.

## ENDOTHELIAL VESSEL FORMATION

**Figure 1.**
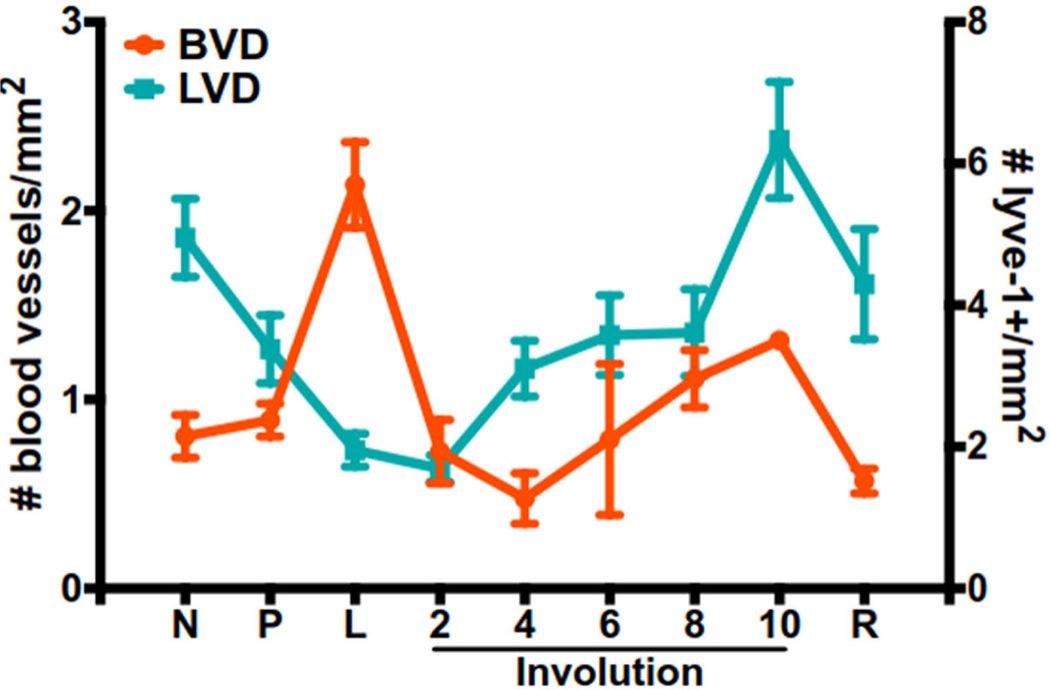
BVD and LVD during mammary gland development. BVD measured as number of blood vessels per millimeter and LVD measured as number of lyve-1 positive vessels per millimeter in nulliparous (N), pregnant (P), lactating (L), involuting (days 2–10) and regressed (R), mammary glands (data adapted from Lyons *et al*.^[16]^ and Ramirez *et al*.^[144]^). BVD: Blood vessel density; LVD: lymphatic vessel density

Blood and lymphatic vessels form two similar, yet distinct, organ systems that assemble into extensive networks throughout the body to support development and survival. Blood vessels provide tissues with oxygen and nutrients, while the primary functions of lymphatic vessels are immune cell trafficking and removal of excess interstitial fluid from tissues. Blood and lymphatic vessels are lined with blood endothelial cells and lymphatic endothelial cells, respectively, and are both surrounded by a thin layer of smooth muscle. Some lymphatic vessels have unique “button-like” junctions that differ from the more continuous “zipper-like” junctions of the blood vasculature and established lymphatic vessels. These specialized junctions are covered with a flap that opens and closes to allow fluid and cells to pass without affecting vascular integrity. Vascular networks are highly dynamic, expanding and retracting as tissues change in response to normal developmental processes or pathologies. Indeed, a widely accepted hallmark of cancer is the ability of tumors to induce angiogenesis, or the development of new blood vessels from existing vasculature^[59]^. Tumors must acquire pro-angiogenic abilities in order to grow beyond 1–2 mm^3[141]^; otherwise, the tumor will die by necrosis or apoptosis^[142,143]^. In rat mammary tissues, we observe an overall net increase in lymphatic vessel density (LVD) during involution when compared to a lactation timepoint^[16]^. In contrast, blood vessel density (BVD) drops dramatically after lactation, suggesting an initial period of regression before increasing in a manner similar to LVD^[144]^. During involution, the highest vasculature densities peak at day 10, followed by a slight decrease in the fully regressed gland [Figure 1]. This is consistent with published studies from our group and others describing increased pro-angiogenic and pro-lymphatic signaling during postpartum involution, and in postpartum tumors compared to non-postpartum controls^[14,16,22,145]^. Interestingly, fibrillary collagen, COX-2, and SEMA7A all have established roles in endothelial vessel formation. Studies using artificial collagen matrices have shown that collagen increases angiogenic responses from endothelial cells by providing the support needed for sustained endothelial cell growth and the formation of endothelial networks^[146]^. Angiogenesis can also be regulated by mechanical stiffness within the small microvessel environment^[147]^, and breast tumors are often stiffer than neighboring normal tissues by up to 6-fold^[84,148]^. For a more comprehensive review on collagen and angiogenesis, see Fang *et al.*^[149]^. These studies demonstrate how the ECM in the TME can modulate vessel formation and alter the tumor’s blood supply.

If a tumor outgrows its blood supply and loses its access to oxygen, it can become hypoxic. Under normal oxygen content conditions, termed “normoxia,” the transcriptional regulator hypoxia-inducible factor-1 (HIF-1) is unable to affect its targets; for further review of this topic, see Masson and Ratcliffe^[150]^. HIF-1 is comprised of two subunits - ARNT/HIF-1β, which is constitutively expressed, and HIF-1α, which is an oxygen-sensitive subunit. During normoxia, HIF-1α is hydroxylated by prolyl hydroxylase domain containing proteins, ubiquitinated by von-Hippel-Lindau protein, and rapidly degraded. During hypoxia, however, HIF-1α is not hydroxylated and degraded, but instead, translocates to the nucleus, heterodimerizes with ARNT, and induces transcription of its target genes. HIF-1 affects a variety of targets, including pro-angiogenic genes like vascular endothelial growth factor-A (VEGF-A)^[151]^. In breast cancer cells, COX-2 can induce inflammation-associated HIF-1 activity, resulting in the expression of pro-angiogenic genes^[48]^. Further, HIF-1 and COX-2 maintain a positive feedback loop, as HIF-1 can also induce expression of COX-2^[152]^. Utilizing NSAIDs to target COX-2 activity can inhibit angiogenesis, demonstrating the potential for therapeutic intervention^[152–154]^. Corroborating our findings that SEMA7A may be involved in COX-2 associated pathways, HIF-1 can directly upregulate SEMA7A in endothelial cells^[155]^. SEMA7A can elicit the release of pro-inflammatory cytokines and cause increased endothelial barrier permeability^[156,157]^. SEMA7A can also promote angiogenesis in a hypoxia-independent manner in murine mammary carcinoma and in the cornea by stimulating macrophages to produce pro-angiogenic molecules, such as CXCL2/MIP-2^[158]^ and VEGF-A^[159]^.

In addition to their roles in angiogenesis of the blood vasculature, collagen, COX-2, and SEMA7A also have known roles in lymphangiogenesis. In breast cancer, increased LVD, lymph node involvement, and lymph vessel invasion are predictive of higher risk of metastasis; further, increased LVD and lymph node metastasis are commonly observed in PPBC^[16]^. While the role of collagen in lymphangiogenesis has not been extensively characterized, one study has shown that collagen I increases lymphangiogenesis and angiogenesis in mouse embryoid bodies under hypoxic conditions^[160]^. Therefore, it is plausible that other fibrillar collagens may contribute to lymphangiogenesis during mammary tumorigenesis. COX-2/PGE2 signaling also promotes production of pro-angiogenic VEGF-A and pro-lymphangiogenic VEGF-C and VEGF-D^[161]^; further, lymphangiogenesis during wound healing is dependent on COX-2 activity^[162]^. Moreover, COX-2 has been implicated in lymphangiogenesis in other cancer types, including cervical and gastric^[163,164]^. We published that inhibition of COX-2 with celecoxib and NSAIDs results in decreased LVD, tumor cell invasion into lymphatics, and metastasis during PPBC^[16]^. For more detailed information on COX-2 and lymphangiogenesis in breast cancer, see Lala *et al*.^[165]^. Finally, SEMA7A has a functional role in lymphatic vessel modulation, as we recently published that SEMA7A promotes tumor-associated lymphangiogenesis via macrophage-mediated lymphatic vessel remodeling during postpartum involution and breast cancer^[57]^. These findings suggest an additional mechanism by which SEMA7A, COX-2, and collagen promote tumor progression and metastasis.

## CLINICAL RELEVANCE/CONCLUSION

**Figure 2.**
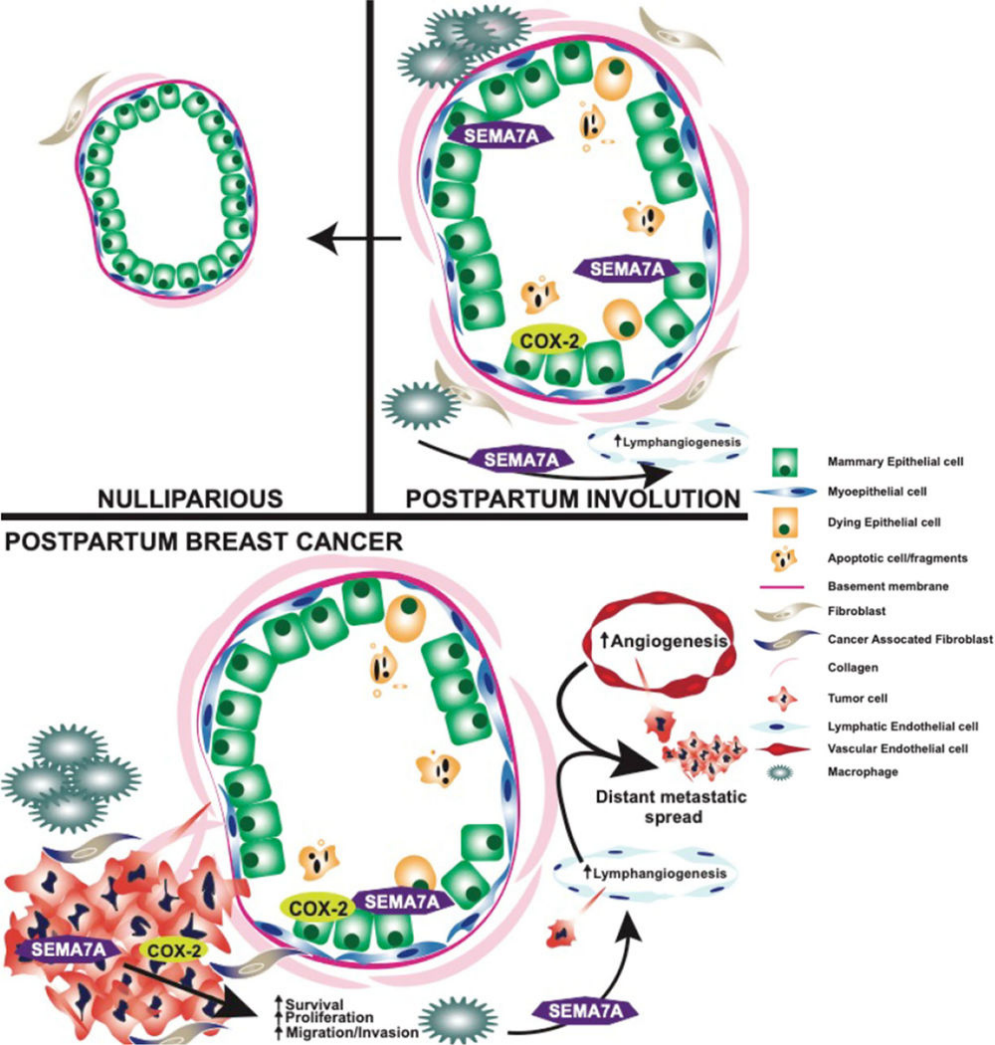
Graphical summary of pro-tumorigenic and metastatic roles of COX-2 and SEMA7A during postpartum involution and postpartum breast cancer. SEMA7A: Semaphorin 7a; COX-2: cyclooxygenase-2

**Figure 3.**
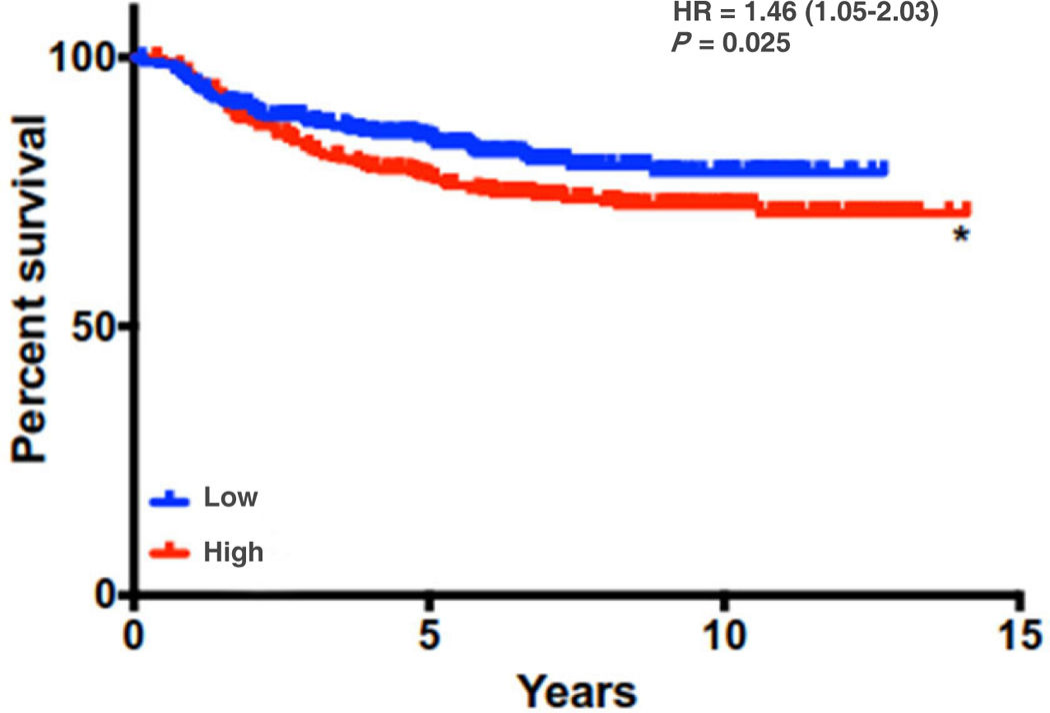
Distant metastasis free survival is decreased with high levels of expression of SEMA7A, COX-2, and COL1A1 signature. DMFS analysis using KmPlot (*n* = 664) *P < 0.05

Women with postpartum breast cancer face a disease that carries three times the rate of metastasis and death relative to women who have never been pregnant and those diagnosed outside of the postpartum window. One physiological event these women have in common is postpartum involution - a process that results in the upregulation and activation of tumor-promotional factors in MECs and the mammary stroma. Identification of the genetic engines that drive PPBC is critical to the development of targeted therapies for postpartum patients. In this review, we have highlighted potential roles for collagen, COX-2, and SEMA7A in driving some of the pro-metastatic aspects of involution [Figure 2]. Previously published results indicate overall survival is generally decreased for breast cancer patients with high collagen, COX-2, and SEMA7A expression, suggesting that these mechanisms are important mediators of breast cancer metastasis^[18,49]^. Interestingly, while individual expression of each molecule does not predict for metastasis using KM Plotter analysis^[166]^, the combination of high SEMA7A, COX-2, and COL1A1 mRNA expression results in significantly decreased distant metastasis free survival for breast cancer patients in this dataset [Figure 3]. Thus, studies rooted in understanding the contributions of postpartum involution associated programs to breast cancer metastasis are likely to also be applicable to general breast cancer metastasis, and perhaps to other cancer types.

Based on the cooperation between SEMA7A, COX-2, and collagen, a multi-targeted therapy to affect the individual molecules and their interplay would likely be more effective than targeting one, alone. The potential of COX-2 as a therapeutic treatment has been investigated in multiple models of cancer. In fact, the COX-2 inhibitor, celecoxib, has been successful in the treatment of a specific type of colorectal cancer - familial adenomatous polyposis - in both adults and children^[167,168]^. Targeting COX-2 in breast cancer, by celecoxib or other NSAIDs, may inhibit tumor cell dissemination by reducing the expression of tumor-promotional collagen. Targeting SEMA7A in conjunction with already established therapies, such as NSAIDs, may also increase the efficacy of these treatments in women with breast cancer. Ideally, the characterization of tumor-promotional factors in the postpartum mammary gland may also lead to preventative therapies aimed at reducing the risk for PPBC. NSAIDs may further represent a safe candidate for preventative therapy during involution via inhibition of COX-2 mediated collagen upregulation and alternative macrophage activation. The topics covered herein highlight both the potential contribution of the SEMA7A/COX-2/Collagen relationship to PPBC, and the importance of PPBC models to the discovery of new molecules and pathways that can be exploited as novel therapeutics.
